# IGFBP-4 and −5 are expressed in first-trimester villi and differentially regulate the migration of HTR-8/SVneo cells

**DOI:** 10.1186/1477-7827-12-123

**Published:** 2014-12-04

**Authors:** Erin J Crosley, Caroline E Dunk, Alexander G Beristain, Julian K Christians

**Affiliations:** Biological Sciences, Simon Fraser University, V5A 1S6 Burnaby, Canada; Research Centre for Women’s and Infants Health, Lunenfeld Tanenbaum Research Institute, Mount Sinai Hospital, Toronto, Canada; Department of Obstetrics and Gynecology, The University of British Columbia, Vancouver, Canada; The Child and Family Research Institute, Vancouver, Canada

**Keywords:** Pappalysins, PAPP-A, PAPP-A2, Insulin-like growth factor-binding proteins, IGFBP-4, IGFBP-5, Trophoblast migration

## Abstract

**Background:**

Adverse gestational outcomes such as preeclampsia (PE) and intrauterine growth restriction (IUGR) are associated with placental insufficiency. Normal placental development relies on the insulin-like growth factors -I and -II (IGF-I and -II), in part to stimulate trophoblast proliferation and extravillous trophoblast (EVT) migration. The insulin-like growth factor binding proteins (IGFBPs) modulate the bioavailability of IGFs in various ways, including sequestration, potentiation, and/or increase in half-life. The roles of IGFBP-4 and −5 in the placenta are unknown, despite consistent associations between pregnancy complications and the levels of two IGFBP-4 and/or −5 proteases, pregnancy-associated plasma protein -A and -A2 (PAPP-A and PAPP-A2). The primary objective of this study was to elucidate the effects of IGFBP-4 and −5 on IGF-I and IGF-II in a model of EVT migration. A related objective was to determine the timing and location of IGFBP-4 and −5 expression in the placental villi.

**Methods:**

We used wound healing assays to examine the effects of IGFBP-4 and −5 on the migration of HTR-8/SVneo cells following 4 hours of serum starvation and 24 hours of treatment. Localization of IGFBP-4, −5 and PAPP-A2 was assessed by immunohistochemical staining of first trimester placental sections.

**Results:**

2 nM IGF-I and -II each increased HTR-8/SVneo cell migration with IGF-I increasing migration significantly more than IGF-II. IGFBP-4 and −5 showed different levels of inhibition against IGF-I. 20 nM IGFBP-4 completely blocked the effects of 2 nM IGF-I, while 20 nM IGFBP-5 significantly reduced the effects of 2 nM IGF-I, but not to control levels. Either 20 nM IGFBP-4 or 20 nM IGFBP-5 completely blocked the effects of 2 nM IGF-II. Immunohistochemistry revealed co-localization of IGFBP-4, IGFBP-5 and PAPP-A2 in the syncytiotrophoblast layer of first trimester placental villi as early as 5 weeks of gestational age.

**Conclusions:**

IGFBP-4 and −5 show different levels of inhibition on the migration-stimulating effects of IGF-I and IGF-II, suggesting different roles for PAPP-A and PAPP-A2. Moreover, co-localization of the pappalysins and their substrates within placental villi suggests undescribed roles of these molecules in early placental development.

**Electronic supplementary material:**

The online version of this article (doi:10.1186/1477-7827-12-123) contains supplementary material, which is available to authorized users.

## Background

Adverse gestational outcomes such as preeclampsia (PE) and intrauterine growth restriction (IUGR) are thought to be caused, at least in part, by deficiencies in processes critical to placental development, including extravillous trophoblast (EVT) invasion. EVT invade the maternal decidua and replace the endothelium of uterine spiral arteries, thus increasing vessel diameter to ensure adequate blood flow required for oxygen and nutrient delivery to the placenta [1, 2]. In both human villous explants and primary trophoblast cultures, insulin-like growth factors I and II (IGF-I and IGF-II, respectively), stimulate trophoblast proliferation and EVT migration [3, 4].

The bioavailability of IGF-I and -II is modulated by six insulin-like growth factor-binding proteins (IGFBPs) [3, 5, 6], with the release of the IGFs generally achieved through proteolysis of the IGFBPs [7]. In addition to reducing the availability of the IGFs, in some contexts IGFBPs increase the half-life of IGFs, concentrate them in particular regions and/or potentiate their effects [8]. Furthermore, IGFBP-1, IGFBP-2, IGFBP-3 and IGFBP-5 all exert IGF-independent effects in a variety of cell models and tissues [3, 6, 9]. For example, IGFBP-1 stimulates migration in an IGF-independent manner in HTR-8/SVneo cells, an immortalized trophoblast cell line commonly used to model EVT migration and invasion [10]. In BeWo cells, an immortalized choriocarcinoma cell line commonly used to model the villous cytotrophoblast, IGFBP-3 inhibits proliferation in an IGF-independent manner [11]. The proteolytic fragments of cleaved IGFBP-3 and −5 have also been shown to have effects in other systems [12–14].

The pappalysins, pregnancy-associated plasma proteins-A and -A2 (PAPP-A and PAPP-A2, respectively), are two IGFBP proteases that have been consistently associated with a range of pregnancy complications. PAPP-A is a protease of IGFBP-4 and −5 that is produced by the placenta [15] and circulates in the maternal blood at high levels during pregnancy. Abnormally low levels of PAPP-A in the first trimester have frequently been associated with increased risk of PE and IUGR [16]. Pregnancy-associated plasma protein-A2 (PAPP-A2) shares 45% amino acid identity with PAPP-A, cleaves IGFBP-5 but not IGFBP-4, and is also produced by the syncytiotrophoblast and released into the maternal circulation during pregnancy [17]. PAPP-A2 levels in the placenta and maternal circulation are higher at term in preeclamptic pregnancies and pregnancies with severe fetal growth restriction [18–21], and PAPP-A2 levels are also elevated in the first-trimester maternal serum of pregnancies that subsequently develop preeclampsia [22].

The mechanistic links between circulating pappalysin levels and pregnancy complications, and whether altered pappalysin expression plays a causal role in placental pathologies, remain unknown. However, whatever role the pappalysins play in placental development and physiology likely involves their IGFBP substrates, IGFBP-4 and −5. While IGFBP-1 is the most abundant IGFBP within the maternal decidua [23] and is relatively well-studied, the roles of IGFBP-4 and −5 in placental development have received little attention.

The purpose of this study was to investigate whether the pappalysins might influence EVT migration and invasion through effects on IGF availability and/or other actions of the IGFBPs. We employed a well-established model of first trimester EVT to examine the effects of exogenous IGFBP-4 and −5 on the migration-stimulating effects of IGF-I and IGF-II. We focused primarily on the inhibition of the migration, but also tested for potentiation and IGF-independent effects. The IGFBPs are known to be expressed in the maternal decidua [23] and therefore are well positioned to regulate EVT invasion. However, to examine whether IGFBP-4 and −5 might also regulate processes within the villi, we examined the expression of these binding proteins in first-trimester villi using immunohistochemistry.

## Methods

### Cell lines and cell culture

HTR-8/SVneo cells, an immortalized cell line that is a well-established model of first trimester human trophoblasts, were obtained from Dr. Charles Graham (Queen’s University, Kingston, ON, Canada) [24, 25]. Cells were cultured in RPMI 1640 medium supplemented with 10% fetal bovine serum, 100 U/mL of penicillin, and 100 U/mL of streptomycin at 37°C in a humidified atmosphere of 5% CO_2_. Cell culture media and reagents were purchased from Life Technologies (Burlington, ON, Canada).

### HTR-8/SVneo cell wounding assay

HTR-8/SVneo cells were seeded at a density of 40,000 cells per well in 24-well polystyrene tissue culture plates and allowed to grow to confluence before serum starving for at least 4 hours. Wounding was performed with a 20 μL pipette tip across the horizontal midsection of each well, and photographs were taken at two points along each wound. The XY coordinates of each point were saved using Simple PCI coordinate mapping software and photographed again 24 hours after treatment (described below). The percentage wound closure was calculated as (1 – (area of wound at 24 hours/area of wound at time 0)) ×100%, as quantified using ImageJ software (see Additional file 1: Figure S1). Recombinant human IGF-I, IGF-II, IGFBP-4 and IGFBP-5 were purchased from R&D Systems (Minneapolis, Minnesota). A dose response experiment ranging from 2–25 nM IGF-I or –II established that 2 nM was sufficient to generate a significant increase in migration of HTR-8/SVneo cells. Dosage in the range of 1–25 nM IGFBP-4 or −5 was expected to have inhibitory effects based on the literature [26] and preliminary experiments. Migration experiments examining the stimulatory effects of the IGFs were performed by treating HTR-8/SVneo cells for 24 hours with either 2 nM IGF-I or IGF-II. Migration experiments examining the inhibitory effects of the IGFBPs were performed by co-treating HTR-8/SVneo cells with either 20 nM IGFBP-4 or IGFBP-5, and one of the two IGFs at 2 nM. Experiments examining IGF-I potentiating effects of low doses of IGFBP-5 were performed across a range of concentrations from equimolar amounts of IGF-I and IGFBP-5, to an 8 fold excess of IGF-I against IGFBP-5 based on previous work with IGFBP-1 [27, 28]. Potentiating effects of IGFBP-4 were not investigated because we know of no evidence that IGFBP-4 has IGF-potentiating effects in other systems [29]. Preliminary experiments examining potential IGF-independent effects of 20 nM IGFBP-4 or 20 nM IGFBP-5 were also performed.

### Immunohistochemistry

Human placental tissue was collected from elective terminations of pregnancies between 5 and 13 weeks of gestation. Sections from a total of 6 different placentae were used. Upon collection, samples were fixed overnight in 4% paraformaldehyde, paraffin-embedded, sectioned (4–5 μm) and mounted. Sections were deparaffinized in xylene and rehydrated with a graded series of alcohol. Antigen retrieval was performed by heating sections in 10 mM citrate buffer for 30 min. Immunohistochemical staining was performed using a horseradish peroxidase- 3-amino-9-ethylcarbazole (HRP-AEC) goat kit (R&D Systems) according to the manufacturer’s instructions. Adjacent sections were incubated with primary polyclonal anti-human antibodies against one of cluster of differentiation 31 (CD31), PAPP-A2, IGFBP-4 or IGFBP-5, all of which were raised in goat (R&D Systems). Immunoreactivity was visualized with AEC and sections were counterstained with hematoxylin. Negative controls were prepared using non-specific goat immunoglobulin G (IgG; R&D Systems) in place of primary antibodies. The use of these samples was approved by the University of British Columbia Children’s and Women’s Research Ethics Board, the Mount Sinai Hospital Research Ethics Board and the Simon Fraser University Research Ethics Board.

### Statistical analyses

Effects of treatments were analyzed in JMP (ver. 10; SAS Institute Inc.) using a general linear model including the effects of treatment and date of experiment. Treatment replicates were distributed across different experimental dates, with replication both within and between days. All experiments contained the appropriate controls (controls, and IGF-I and/or –II alone) to allow for comparison across dates (with similar sample sizes for treatments performed on the same day).

## Results

### IGF-I, IGF-II and HTR-8/SVneo migration

**Figure 1:**
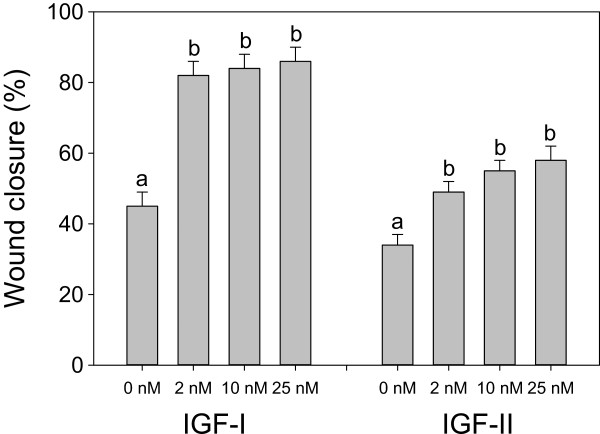
**Effects of IGF-I and IGF-II dose on migration of HTR-8/SVneo cells.** IGF-I and –II experiments were conducted separately and therefore have different control values. Values are mean ± standard error of the mean, and values with different superscript letters within an experiment are significantly different according to Tukey’s HSD test (overall variation in IGF-I experiments: F_3, 20_ = 28.65, P < 0.0001, N = 6 per treatment; IGF-II experiments: F_3, 43_ = 12.86, P < 0.0001, N = 6–14 per treatment).

**Figure 2:**
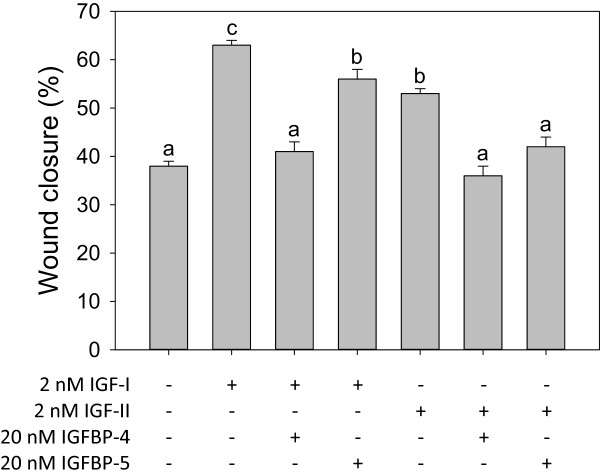
**Effects of IGF-I, IGF-II, IGFBP-4 and IGFBP-5 on migration of HTR-8/SVneo cells.** Values are mean ± standard error of the mean, and values with different superscript letters are significantly different according to Tukey’s HSD test (overall variation among treatments: F_6, 249_ = 38.11, P < 0.0001, N = 24–62 per treatment).

Preliminary experiments with 2 nM, 10 nM or 25 nM of either IGF-I or IGF-II showed a significant stimulatory effect of 2 nM on HTR-8/SVneo cells (Figure 1). Treating HTR-8/SVneo cells with either IGF-I or IGF-II at 2 nM significantly increased migration compared with controls, with IGF-I increasing migration significantly more than IGF-II (Figure 2).

### IGFBP-4, IGFBP-5 inhibit effects of IGF-I and IGF-II on HTR-8/SVneo migration

**Figure 3:**
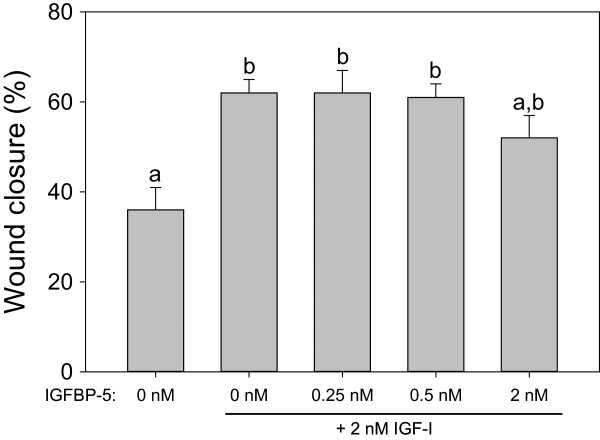
**Effects of low doses of IGFBP-5 on migration of HTR-8/SVneo cells.** Values are mean ± standard error of the mean, and values with different superscript letters are significantly different according to Tukey’s HSD test (overall variation among treatments: F_4, 42_ = 4.38, P < 0.0048, N = 6–14 per treatment).

While IGFBP-4 and −5 both significantly reduced the effects of 2 nM IGF-I, 20 nM IGFBP-4 almost completely blocked the effects of 2 nM IGF-I, whereas 20 nM IGFBP-5 did not (Figure 2). While 2 nM IGF-I increased wound closure to 63%, co-treatment with 20 nM IGFBP-4 reduced wound closure to 41% compared with 38% in controls, but co-treatment with 20nM IGFBP-5 had a lesser effect, reducing IGF-I-mediated wound closure to an intermediate level (56%). Either 20 nM IGFBP-4 or 20 nM IGFBP-5 completely blocked the effects of 2 nM IGF-II (Figure 2). The migration-stimulating effects of IGF-II (wound closure 53%) were completely blocked by 20 nM IGFBP-4 (wound closure 36%) or 20 nM IGFBP-5 (wound closure 42%); neither IGFBP treatment was significantly different from controls (38%). There was no significant effect of IGFBP-4 or IGFBP-5 alone at 20 nM in preliminary experiments, suggesting an absence of IGF-independent effects (data not shown). Moreover, there was no significant potentiation effect of low doses of IGFBP-5 with 2 nM IGF-I (Figure 3).

### Immunoreactivity for IGFBP-4, IGFBP-5 and PAPP-A2 in the syncytiotrophoblast

**Figure 4:**
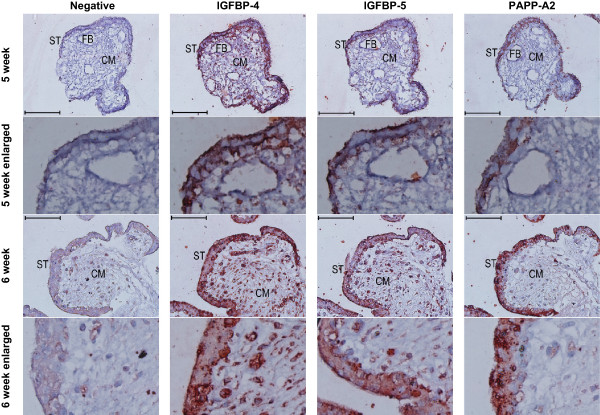
**Immunoreactivity (red colour) against IGFBP-4, −5, and PAPPA2 in the syncytiotrophoblast of first trimester placental villi.** Serial cross sections of placental villi at 5 and 6 weeks of gestation (7 and 13 weeks not shown) were stained for IGFBP-4, IGFBP-5, and PAPP-A2. Negative controls used non-specific goat IgG in the place of the primary antibodies. FB = fetal blood vessel, CM = chorionic mesoderm, ST = syncytiotrophoblast. Scale bars denote 100 μm.

The presence of IGFBP-4, IGFBP-5 and PAPP-A2 was examined in serial sections of placental villi at 5, 6, 7 and 13 weeks of gestation. In addition to the non-specific goat IgG negative control, staining against CD31 was used as a negative control for immunoreactivity in the syncytiotrophoblast, since staining was only expected in the endothelium of fetal vessels. Across all gestational ages, IGFBP-4 and −5 showed localization within the syncytiotrophoblast, with stronger immunoreactivity observed for IGFBP-4 than IGFBP-5 (Figure 4). IGFBP-4 and −5 also appeared in the chorionic mesoderm. PAPP-A2 in contrast, showed little to no immunoreactivity in the chorionic mesoderm at 5 and 6 weeks, but did show strong immunoreactivity in the syncytiotrophoblast (Figure 4).

## Discussion

To investigate the potential mechanism underlying the associations between levels of PAPP-A and PAPP-A2 and pregnancy complications such as PE and IUGR, we studied the effects of pappalysin substrates IGFBP-4 and −5 on IGF-I and -II in a model of EVT migration. We also examined the location and timing of IGFBP-4 and −5 expression to determine whether the pappalysins might also influence processes occurring within the villi early in pregnancy.

Consistent with previous findings in various models of first trimester human EVT [30–36], both IGF-I and IGF-II at 2 nM significantly stimulated migration of HTR-8/SVneo cells in a cell-wounding assay. Furthermore, IGF-I had greater stimulatory effects than IGF-II at this concentration. The difference in effects of IGF-I and –II may be due to the presence of both type 1 and type 2 IGF receptors (IGF1R and IGF2R) in HTR-8/SVneo cells [26, 36], and the higher binding affinity of IGF1R for IGF-I than for IGF-II [37].

IGFBP-4 and −5 showed different levels of IGF inhibition. IGFBP-4 was able to block the migration-stimulating effects of both IGF-I and IGF-II to control levels. In contrast, IGFBP-5 was able to inhibit the migration-stimulating effects of IGF-II to control levels, but only partially inhibited IGF-I. While previous work has shown IGFBP-5 blocks IGF-II stimulation of migration in a cell line model of EVT [26], this is to our knowledge the first data regarding the effects of exogenous IGFBP-4 on trophoblast migration. IGFBP-4 has however been found to influence migration and invasion in cancer studies, with inhibitory or stimulatory effects on migration depending on the model examined [38–42]. Similarly, the effects of IGFBP-5 on cellular proliferation and invasion in breast cancer studies appear to be cell line dependent [43]. The inhibitory effect of IGFBP-4 on trophoblast migration may, at least in part, underlie the association between elevated circulating levels of IGFBP-4 in early pregnancy and the subsequent development of fetal growth restriction [44].

There was no evidence of potentiation of the effects of IGF-I by low doses of IGFBP-5, either because no such effect exists *in vivo*, or because IGF potentiation requires interaction between IGFBP-5 and the extracellular matrix [45]. Similarly, the lack of IGF-independent effects of IGFBP-4 or −5 in our experiments may be due to a real absence of effects *in vivo*, to the absence of as-yet-uncharacterized cell surface IGFBP-4 and −5 receptors in HTR-8/SVneo cells [29], or to a requirement for interaction with the extracellular matrix [45] or proteolysis of the IGFBPs [12, 14].

IGF-I and -II have different primary sources in the placenta. IGF-II is strongly expressed by EVT and syncytiotrophoblast in the human placenta [3, 4, 23], whereas the predominant source of IGF-I in the placenta is the maternal circulation, as it is only weakly expressed by the placenta [4, 23]. Low levels of IGF-I mRNA relative to IGF-II mRNA have been detected in the cytotrophoblast, mesodermal core and endothelium of human placental villi (in first, second and third trimesters), but not in EVT [30, 46, 47]. IGF-I has been detected less consistently at the protein level. In one study, IGF-I protein was undetectable in human placental lysates [48], and in another IGF-I protein was detected in first trimester villous cytotrophoblasts, but at much lower levels than IGF-II [47].

A number of observations from previous work and this study suggest that PAPP-A and PAPP-A2 play very different roles in normal placental development and disease, which may explain why PAPP-A and PAPP-A2 show contrasting levels in relation to adverse pregnancy outcomes (i.e., PAPP-A being down-regulated and PAPP-A2 being upregulated in association with pregnancy complications [16]). PAPP-A is a protease of both IGFBP-4 and IGFBP-5, whereas PAPP-A2 only proteolyzes IGFBP-5. Therefore our data suggest that PAPP-A2 has less of a stimulatory effect on EVT migration than PAPP-A, as IGFBP-5 shows less inhibition of IGF-I, which is more potent than IGF-II. Furthermore, PAPP-A may have a stronger effect modulating the availability of IGF-I from the maternal circulation than PAPP-A2.

PAPP-A and PAPP-A2 are expressed by the syncytiotrophoblast as well as invasive EVT [49, 50]. It was previously thought that the only location of expression of IGFBP-4 and −5 in the first trimester was the maternal decidua, since IGFBP-5 had only been observed in villi in the second and third trimester [23]. More recently, IGFBP-4 immunoreactivity has been observed in the chorionic mesoderm of placental villi at 10–13 weeks [44]. Here, we show that IGFBP-4 and −5 are present in the syncytiotrophoblast of placental villi as early as 5 weeks of gestation. PAPP-A and PAPP-A2 may therefore influence aspects of early placental development in addition to the EVT invasion of the decidua and remodeling of the spiral arteries, e.g., cytotrophoblast proliferation or fusion with the syncytiotrophoblast. While we do not know whether the placentae we sampled were from pregnancies that would have gone on to develop pregnancy complications, we observed consistent results in 6 different placentae, 3 of which were at a gestational age of 7 weeks or less. It is very unlikely that none of these early placentae were from a healthy, uncomplicated pregnancy.

## Conclusions

IGFBP-4 and −5 have differing effects on IGF-I and -II in an *in vitro* model of EVT migration. Given that PAPP-A and PAPP-A2 show contrasting patterns of regulation in adverse pregnancy outcomes, we propose that the stimulatory effects of PAPP-A2 on EVT migration may be less than those of PAPP-A, and that PAPP-A2 may function principally to modulate IGF-II produced locally in the placenta via its effects on IGFBP-5. Moreover, given the lack of IGF-independent and potentiating effects of the pappalysin substrates, we propose that the effects of the pappalysins are principally stimulatory on migration. This hypothesis suggests that low PAPP-A levels in early pregnancy may contribute to placental pathology, whereas high PAPP-A2 levels in pregnancies that go on to develop complications may represent a compensatory response [51, 52].

## Electronic supplementary material

Additional file 1: Figure S1: HTR-8/SVneo cell wounding assay. (DOC 524 KB)

## Abbreviations

AEC: 3-amino-9-ethylcarbazole
CD31: Cluster of differentiation 31
EVT: Extravillous trophoblast
HRP: Horseradish peroxidase
IGF: Insulin-like growth factor
IGF1R: Insulin-like growth factor 1 receptor
IGF2R: Insulin-like growth factor 2 receptor
IGFBP: Insulin-like growth factor binding protein
IgG: Immunoglobulin G
IUGR: Intrauterine growth restriction
PAPP-A: Pregnancy-associated plasma protein–A
PE: Preeclampsia.
