# Supplementary material for: IGFBP-4 and −5 are expressed in first-trimester villi and differentially regulate the migration of HTR-8/SVneo cells
Source: Reprod Biol Endocrinol. 2014 Dec 4;12:123. doi: 10.1186/1477-7827-12-123 (PMC4271501; doi:10.1186/1477-7827-12-123)
Supplement: Supplementary file 1 — Additional file 1: Figure S1: HTR-8/SVneo cell wounding assay. (DOC 524 KB) [file 12958_2014_1290_MOESM1_ESM.doc]

| Time 0 hours | Time 24 hours |
| --- | --- |
| A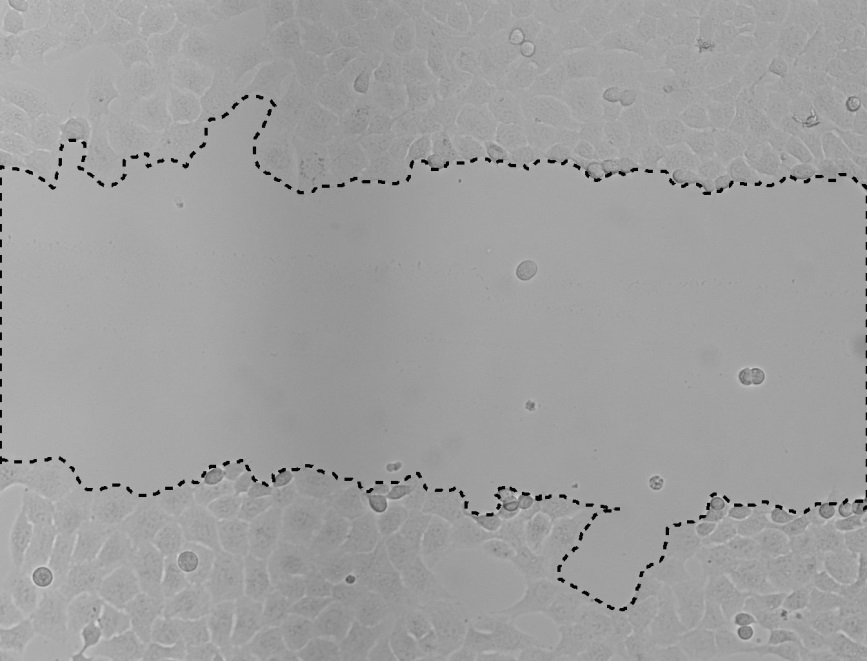 | B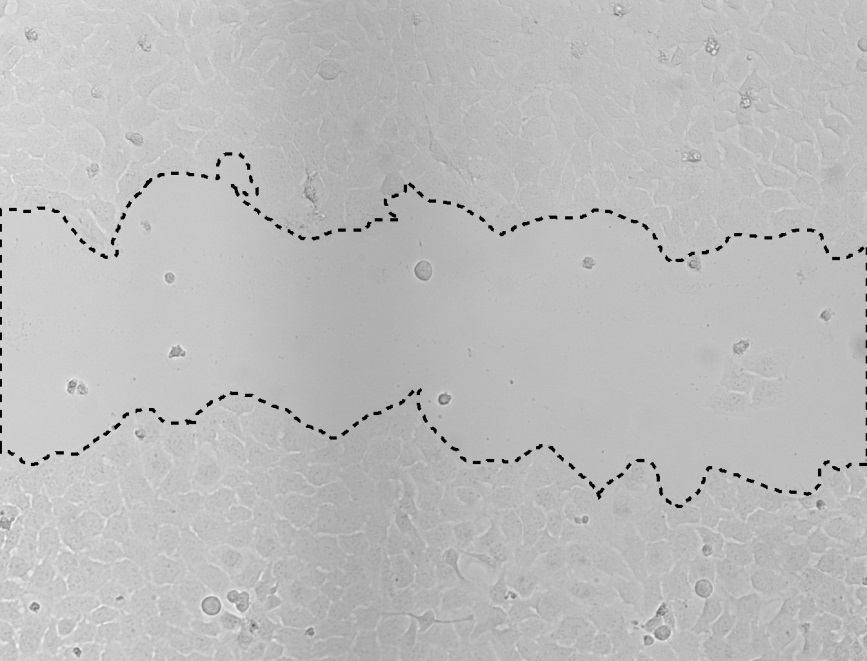 |
|  |  |
| C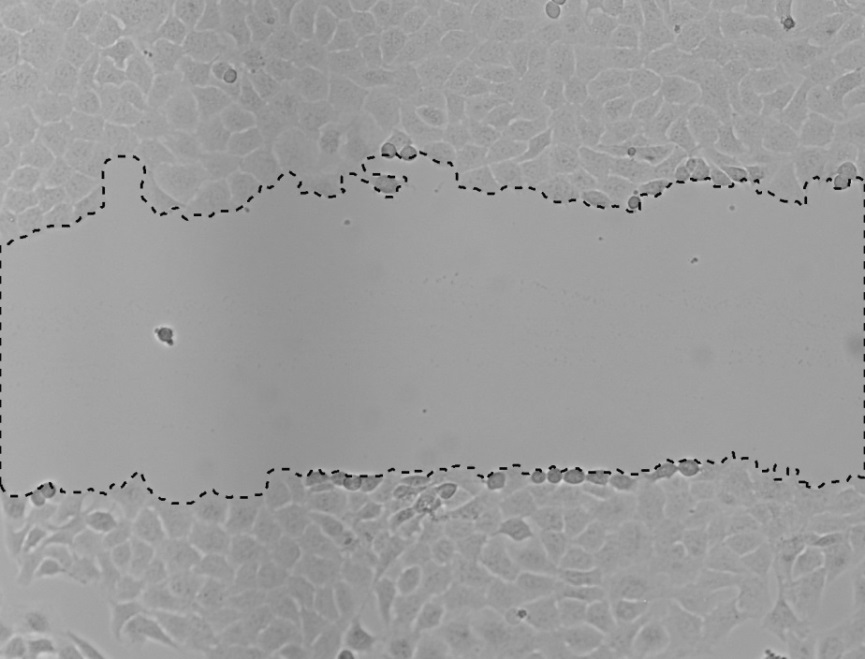 | D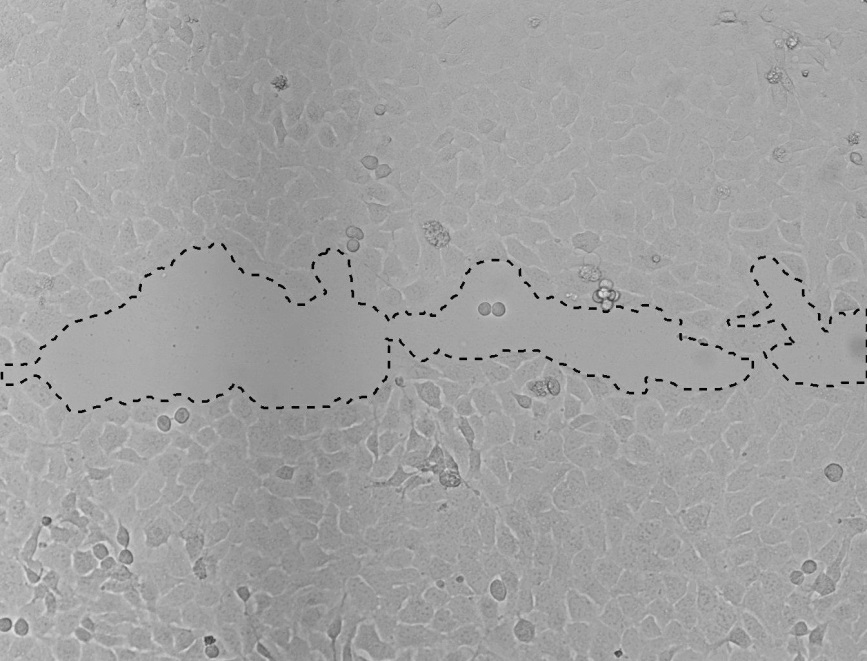 |

**Supplemental Figure S1** - **HTR-8/SVneo cell wounding assay**

HTR-8/SVneo cells at time 0 (A & C) and 24 hours later following treatment with either vehicle (B) or 2 nM IGF-I (D). Dashed line shows outline used to measure area of wound.
